# Acetalax and Bisacodyl for the Treatment of Triple-Negative Breast Cancer: A Combined Molecular and Preclinical Study

**DOI:** 10.1158/2767-9764.CRC-24-0435

**Published:** 2025-02-28

**Authors:** William C. Reinhold, Elisabetta Marangoni, Fathi Elloumi, Remi Montagne, Sudhir Varma, Yanghsin Wang, Keyvan Rezai, Ludivine Morriset, Ahmed Dahmani, Rania El Botty, Léa Huguet, Makito Mizunuma, Naoko Takebe, Samuel Huguet, Augustin Luna, Yves Pommier

**Affiliations:** 1 Developmental Therapeutics Branch, Center for Cancer Research, National Cancer Institute, NIH, Bethesda, Maryland.; 2 Translational Research Department, Institut Curie, PSL University, Paris, France.; 3 CBIO-Centre for Computational Biology, Institut Curie, INSERM, Mines ParisTech, Paris, France.; 4 HiThru Analytics LLC, Princeton, New Jersey.; 5 ICF International Inc., Fairfax, Virginia.; 6 Institut Curie, Département de Radio-Pharmacologie, Saint-Cloud, France.; 7 Computational Biology Branch, National Library of Medicine, NIH, Bethesda, Maryland.

## Abstract

The recalcitrant nature of triple-negative breast cancer (TNBC) calls for novel treatments. Acetalax (oxyphenisatin acetate) and bisacodyl, which have been and are extensively used orally as laxatives, have recently been reported to exhibit selective antiproliferative activity when incubated with cancer cells and xenograft models. Here, we show that they are both preferentially active in TNBC cell lines, and that their activity patterns overlap across the 1,000 cell lines of the Sanger database but are unique and distinct from the standard chemotherapies. Multivariate transcriptomic analyses demonstrate that expression of the ion transporter TRPM4 and two other cell surface genes predict the activity of both drugs. *In vivo* testing shows that both Acetalax and bisacodyl induce complete regression of different TNBC patient-derived xenografts. Acetalax pharmacokinetics shows a 5.8-hour terminal half-life and drug penetration in TNBC tumors and brain.

**Significance:**

Acetalax and bisacodyl represent a prospective novel drug mechanism-of-action type, affect mitochondrial function and affect tumor growth *in vivo*. Their activity may be predicted by *TRPM4* but with more accuracy adding other genes in multivariate analysis for triple negative breast cancer (TNBC). Acetalax has a biphasic mean half-life of 5.8 hours.

## Introduction

Triple-negative breast cancer (TNBC) accounts for 10% to 15% of breast cancers and is a highly malignant subtype characterized by high invasiveness, high metastatic potential, poor prognosis, and limited treatment options due to an absence of many well-defined targetable alterations (1–3). Only a small portion of patients with TNBC (∼20%) respond well to standard therapies (i.e., surgery, radiation, and chemotherapy; ref. 4).

There is significant interest in repurposing approved therapies for treating refractory cancers (5, 6). Acetalax and bisacodyl are relatively old drugs, patented in 1959 and 1956, respectively, for use as laxatives when taken orally. Acetalax has been marketed under the names of Contax, Acetophenolisatin, Brocatine, Diphesatin, and Acetalax. Bisacodyl has gone under the names of Dulcolax, Durolax, Muxol, Fleet, Nourilax, Alophen, and Correctol. The two drugs are stimulant laxatives with local secretory action, leading to increased water content in the stool (7). Although bisacodyl remains in use today, Acetalax was withdrawn in most countries in the early 1970s because of liver damage, usually occurring when the drug was taken for at least 6 months and more usually for 2 years (Inxight Drugs, https://drugs.ncats.io/drug/3BT0VQG2GQ). The use of either of these in cancer would be examples of drug repurposing for the treatment of TNBC.

There has been some indication for the use of these drugs in cancer. Bisacodyl has been reported to target quiescent glioma stem cells, induce *in vivo* tumor shrinkage, and increase survival in glioblastoma models (8). Acetalax has been reported to have multiple effects in breast cancer cell lines, including triggering a cell starvation response and autophagy (9).

TNBC cells surviving neoadjuvant chemotherapy have been proposed to have a unique therapeutic dependency on mitochondrial oxidative phosphorylation (OXPHOS) levels, possibly associated with mitochondrial morphology and fragmentation with elongated mitochondria associated with higher levels of OXPHOS (10). The study suggests targeting mitochondrial metabolism in TNBC (10).

In recent work, we identified sensitivity of TNBC cell lines to Acetalax based on its testing in the NCI-60 (patent EP 19707221.8 | NIH E-041-2018-0-EP-03 | MBHB 21-0947-WO-EP). As a follow-up, we conducted large-scale drug screenings of both Acetalax and bisacodyl in the Genomics of Drug Sensitivity in Cancer (GDSC) collection of cell lines, and incorporated these data into our CellMinerCDB web application (https://discover.nci.nih.gov/rsconnect/cellminercdb/; refs. 6, 11). Comparison of the GDSC and Developmental Therapeutics Program (DTP) screenings showed the activities of both Acetalax (r = 0.80; *P* = 1.1E−10) and bisacodyl (r = 0.84; *P* = 9.0E−13) to be highly correlated across platforms. Both platforms displayed outstanding activity for both drugs in two of the three NCI-60 TNBC cell lines (bioRxiv 2018.292904; ref. 6), with approximately one third of the GDSC TNBCs appearing sensitive. Comparison with other FDA-approved anticancer drugs indicated outstanding activity for both drugs in two of the three NCI-60 TNBC cell lines in addition to having a good range across cell lines from all cancers. We suggested that Acetalax and bisacodyl might be used for treating TNBC as repurposed drugs (6) and showed that the transient receptor potential cation channel subfamily M member 4 (TRPM4) protein is the target and biomarker for Acetalax, with the protein being present in sensitive but not resistant TNBCs (12). In addition, we reported that Acetalax- and bisacodyl-sensitive TNBC cell lines undergo multiple alterations, including (i) increased cell permeability, (ii) cellular swelling, (iii) nuclear swelling, (iv) membrane blebbing, (v) decreased membrane potential, and (vi) ATP depletion (12). These characteristics have previously been described as occurring in oncosis, a cell death process distinct from apoptosis (12–15).

In the current study, we first use the GDSC cancer cell lines to provide evidence that Acetalax and bisacodyl have the characteristics of a novel mechanism-of-action drug class, which works preferentially well in the recalcitrant TNBC. We then proceeded with efficacy, pharmacokinetics, and pharmacodynamics in patient-derived xenografts (PDX). We determine the rate of drug breakdown in the blood and the rate of entry into the liver, kidney, brain and tumor. By characterizing gene sets associated with the drug activities, we identify the mitochondrial and epithelial–mesenchymal functional categories as being significantly associated with Acetalax and bisacodyl response, and by doing a combination of single and multivariate analyses, we further characterize prospective drug biomarkers. We demonstrate complete response to the drugs *in vivo* similar to the sensitive response seen in cell lines, implying the models are consistent and informative and the drug has prospective clinical application.

## Materials and Methods

### CellMinerCDB and R Studio use in characterization of Acetalax and bisacodyl in cancer cell lines

All cell line data scatter plots were generated using the Genomics and Pharmacology Facility (RRID: SCR_025664) CellMinerCDB using “Univariate Analysis\Plot Data” tabs (https://discover.nci.nih.gov/rsconnect/cellminercdb/, RRID: SCR_025649). All scatter plots generated by CellMinerCDB will use the same variable selection of cell line subgroups and coloring. The drug activity density plots are derived from the activities downloaded from “CellMinerCDB\Metadata\GDSC-MGH-Sanger\act: Drug Activity [−log_10_(IC_50_M)]”. These activity data were both transformed into *z*-scores (across all cell lines) and then plotted using The R Project for Statistical Computing version 3.8.6 (and for all subsequent R Project references, RRID: SCR_001905). For all cell line work, the National Service Center (NSC) number for bisacodyl is 614826 and the NSC for Acetalax is 59687 (both from the DTP, RRID: SCR_003057). The principal component analysis (RRID: SCR_014676) and scatter plot were created in R Studio using the drug activity *z*-scores as input. The hierarchical clustering of FDA-approved drug activities was generated in R.

### Comparison of Acetalax activity with CRISPR knockdown patterns in TNBC using gene set enrichment analysis

CRISPR gene survival values are reported as CERES scores, a *computational method to estimate gene dependency levels from CRISPR/Cas9* essentiality screens. The correlation with Acetalax was generated using CellMinerCDB (https://discover.nci.nih.gov/rsconnect/cellminercdb/) by selecting “Univariate Analyses\Compare Patterns”, comparing the “*x*-axis cell line set GDSC\Drug activity\Acetalax” to the “*y*-axis cell line set Achilles project (CRISPR data)\To include Breast Triple Negative” resulting in data for 18,121 genes. These data were ranked by correlation (high to low for all cell lines with data) and submitted to gene set enrichment analysis (GSEA, RRID: SCR_003199) using pre-ranked analysis imported into the (GSEA, https://www.gsea-msigdb.org/gsea/index.jsp, version 4.3.2, RRID: SCR_003199) web application using set minimum and maximum sizes of 5 and 500 and a classic scoring scheme to determine enriched functional categories. For the gene sets, we selected the collections H.all, C2.all, C5.all, C6.all, and C7.all. Binomial enrichment was calculated using the R Project for Statistical Computing.

### Comparison of Acetalax activity with transcript expression patterns in TNBC cell lines using GSEA

GSEA (version 4.3.2) was used for the assessment of the five most Acetalax-sensitive cell lines HCC2157 (RRID: CVCL_1261), MRK-nu-1 (RRID: CVCL_1428), HDQ-P1 (RRID: CVCL_2067), HCC70 (RRID: CVCL_1270), and MDA-MB-468 (RRID: CVCL_0419) compared with the five most Acetalax-resistant cell lines CAL-120 (RRID: CVCL_1104), MDA-MB-231 (RRID: CVCL_0062), DU-4475 (RRID: CVCL_1183), HCC1937 (RRID: CVCL_0290), and CAL-51 using GDSC microarray transcript expression (RRID: SCR_025383). For the bisacodyl assessment, the five most bisacodyl-sensitive cell lines (the same as for Acetalax) were compared with five most bisacodyl-resistant cell lines [CAL-120, MDA-MB-231, DU-4475, CAL-85-1 (RRID: CVCL_1114), and MFM-223 (RRID: CVCL_1408)]. These data were analyzed for enriched functional categories using the GSEA algorithm with default parameters of (i) a set minimum size of 15, (ii) a set maximum size of 500, (iii) the signal2noise metric, and (iv) weighted scoring schema. For these gene sets, we selected the following collections: H.all, C2.all, C5.all, C6.all, and C7.all.

### PDXs and *in vivo* preclinical assays

Human breast tumor fragments were obtained with patients’ written informed consent (Institute Curie, RRID: SCR_000959). *In vivo* experimental procedures were approved by the Institutional Animal Care and French Committee (project authorization no. 02163.02). To establish PDX models, tumor fragments were removed during surgery of female patients with breast cancer and grafted into the interscapular fat pad of 8 to 12-week-old female Swiss nude mice under anesthesia, as previously detailed (16–18).

For efficacy studies, Acetalax and bisacodyl were given by intraperitoneal injection 5 days/week at different doses (100, 200, and 300 mg/kg). Carboplatin, paclitaxel, and capecitabine were given at 90 mg/kg (days 1 and 22), 25 mg/kg weekly, and at 540 mg/kg/day (5 days/week), respectively. Adriamycin (doxorubicin), docetaxel, cisplatin, and cyclophosphamide were administered by the intraperitoneal route at doses of 2, 20, 6, and 100 mg/kg, respectively, every 3 weeks.

Treated groups included between four and five mice. When tumors reached a volume of 60 to 200 mm^3^, mice were individually identified and randomly assigned to the control or treated groups and the treatments were started. Treatments were administered over 6 weeks or less if tumor volumes reached the ethical size (2,000 mm^3^). Maximal tumor size/burden was not exceeded.

Tumor growth was evaluated by measurement of two perpendicular diameters of tumors with a caliper twice per week. Individual tumor volumes were calculated as V = a × b^2^/2, with a being the largest diameter and b the smallest. Tumor volumes were reported for the initial volume as relative tumor volume. Percent change in tumor volume was calculated for each tumor as (Vf − V0/V0) × 100, in which V0 = initial volume (at the beginning of treatment) and Vf = final volume (at the end of treatment). Tumor regression (R) was defined as a decrease in tumor volume of at least 50% when taking the baseline tumor volume as reference; at least a 35% increase in tumor volume identified progressive disease (PD); and responses that were between +35 and −50% were considered as stable disease (SD).

### RNA-seq analysis of PDX models

Total RNA was extracted from breast tumor xenograft samples by using the miRNeasy Mini kit (#217004, Qiagen) according to the manufacturer’s procedure, treated by RNase-Free DNase (#79254, Qiagen), quantified, and controlled for quality using a 2100 Bioanalyzer (Agilent, RRID: SCR_018043).

RNA sequencing (RNA-seq) libraries were prepared from 500 ng of total RNA using the Illumina TruSeq Stranded mRNA Library preparation kit which allows strand-specific sequencing. A first step of polyA selection using magnetic beads was done to address sequencing specifically on polyadenylated transcripts. After fragmentation, cDNA synthesis was performed and the resulting fragments were used for dA-tailing, followed by the ligation of TruSeq indexed adapters (Unique Dual Indexing strategy). cDNA libraries were generated using 13 cycles of PCR amplification. After qPCR quantification using the KAPA library quantification kit (Roche), sequencing was carried out on the NovaSeq 6000 instrument (RRID: SCR_016387) from Illumina based on a 2 × 100 cycle mode (paired-end reads, 100 bases) to obtain around 100 million clusters (200 million raw paired-end reads) per sample.

To avoid mouse RNA contamination, reads were analyzed with xengsort (v1.1.0; ref. 19) using human genome hg38 and mouse genome mm10 as references. Human reads were then aligned with human genome reference (hg38) and annotated with the human GENCODE (v38, RRID: SCR_014966) database using the STAR tool (v.2.7.6a; ref. 20). The table of counts per human GENCODE feature was obtained with STAR. Batch effect correction was performed with ComBat_Seq from the package sva (v3.46.0) using the responder status as the biological factor of interest (21). The count table was normalized using method TMM from the R package edgeR (v3.38.4; ref. 22). Differential gene expression was computed with limma (v3.52.4) and corrected for multiple testing using an FDR of 0.05 (23). Genes were ranked by decreasing log fold-change and used to perform GSEA with clusterProfiler (v4.4.4, RRID: SCR_016884). The Gene Ontology (RRID: SCR_002811) and MsigDB (RRID: SCR_016863) databases were used for querying the collections H.all, C2.all, C5.all, C6.all, and C7.all.

GSEA comparisons of RNA-seq transcript levels of PDX tumors from five mice with Acetalax or bisacodyl treatment at 300 mg/kg were compared with five mice without tumor fragments from patient HBCx-185. For both conditions, the count table was normalized using method TMM from the R package edgeR v3.38.4. Then differential gene expression was computed with package limma v3.52.4. Average gene activation or suppression in treated versus untreated tumors was ranked by decreasing log fold-change and used to perform GSEA using package clusterProfiler v4.4.4 to assess functional pathways affected by the drugs.

### Exploration of genes predictive of Acetalax and bisacodyl activity

For the exploration of models for Acetalax and bisacodyl activity prediction, we first developed a list of 222 genes with both biological relevance and a fourfold minimum level of variation within the 22 TNBC cell lines with Acetalax data. CellMinerCDB was used to determine the correlations between the Acetalax activity and gene transcript expression for the TNBC subset, as well as to generate scatter plots. All genes were selected based on a combination of low *P* value and biological consideration. For each subsequent gene addition, we used the same 222 gene set and determined the multivariate linear regression *P* values (calculated in R). To determine the true *P* value, we used our CellMinerCDB multivariate analysis web tool with 10× cross validation.

Correlations between tumor size and the gene expression were obtained by applying the Spearman correlation test using R base function “cor.test()”. Tumor size was expressed as the percentage of their volume at the time of treatment. For each group of tumor-grafted mice, the median volume was selected. Gene expression corresponded to the TMM-normalized log of RNA-seq counts.

### Cell line authentication

No cell lines were obtained for this study. The cell line work referred to was done at the GDSC (https://www.cancerrxgene.org/, RRID: SCR_011956) and DTP (https://dtp.nci.nih.gov, RRID: SCR_003057). Cell authentication and *Mycoplasma* testing were done by those institutes.

### Pharmacokinetic analysis of Acetalax

For the pharmacokinetic experiment, blood and tissue samples were collected from HBCx-158 xenografts at different time points after Acetalax administration given on days 1 and 4. The plasma and tissue concentration of Acetalax and its metabolite were determined using an ultra high-performance liquid chromatography-tandem mass spectrometry (UHPLC-MS/MS) method. The calibration ranges for both molecules were 2.00 to 200 and 25.00 to 10,000 ng/mL in plasma and in tissues, respectively.

### Toxicology

In the absence of formal toxicology, we note that previously a simple toxicity assessment was done for Acetalax by DTP using visual observation and body weight monitoring following administration of single intraperitoneal doses of the compound at 100, 200, and 300 mg/kg in xenografts. The mice were observed for adverse effects for 14 days after dose, with no toxicity being noted (9). The current study using Acetalax or bisacodyl provides supportive observations for a lack of obvious toxicity in mice.

### Data availability

The drug activities, CRISPR, transcript, and epithelial–mesenchymal transition (EMT) cell line data used in this study are available at CellMinerCDB. The PDX RNA-seq data are deposited in the European Genome-phenome *Archive* (*EGA*, https://ega-archive.org/). The study number is EGAS50000000618 and dataset numbers are EGAD50000000875 and EGAD50000000876. The *in vivo* tumor response and pharmacokinetic data are detailed within this article.

## Results

### Cell line results

**Figure 1:**
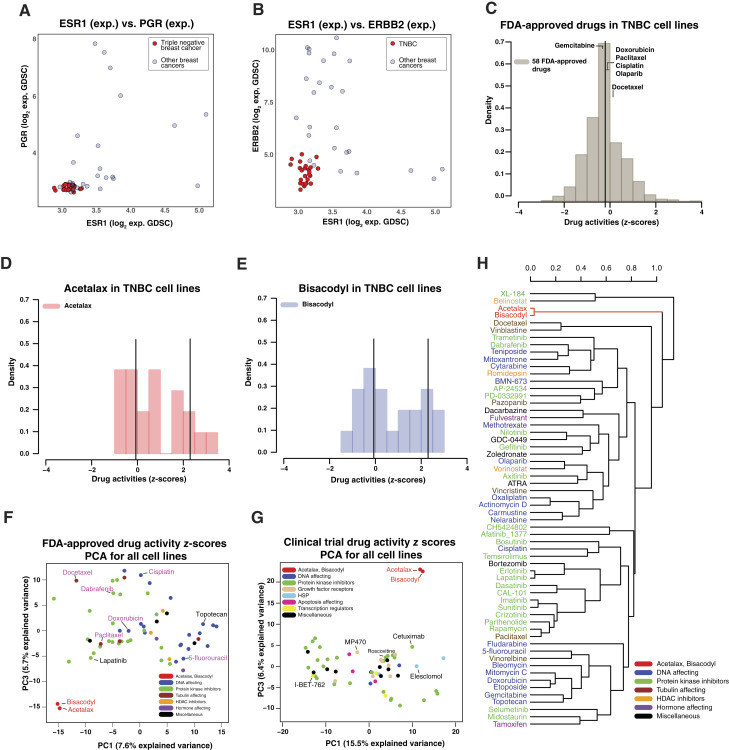
TNBC cell line determination and Acetalax and bisacodyl drug characterization. **A,** Transcript-level scatter plot of estrogen receptor (*ESR1*, *x*-axis) vs. progesterone receptor (PGR, *y*-axis). **B,** Transcript-level scatter plot of *ESR1* (right plot, *x*-axis) vs. *ERBB2* (*y*-axis). For both **A** and **B**, the transcript measurements are from GDSC microarray, the circles are cell lines with red being TNBC and blue the other breast cancer cell lines. For the CDB scatter plot details, “Select Tissues\To include\Breast” was used to limit the cell lines and “Show color”\“Breast Triple negative” to make the triple negatives red. All scatter plots referring to CellMinerCDB will use the same approach with variable selection of cell line subgroups and coloring. **C,** Density plots of drug activity *z*-score comparisons in the 22 TNBC cell lines in GDSC of 58 FDA-approved drugs (gray). The mean values for several drugs used clinically for TNBC are included for reference. **D,** Density plots of drug activity *z*-score comparisons in the 22 TNBC cell lines in GDSC of Acetalax (red). **E,** Density plots of drug activity *z*-score comparisons in the 22 TNBC cell lines in GDSC of bisacodyl (blue). The *x*-axis for **C–E** is the drug activities (*z*-scores). The height of the bars represents the proportion of cells within the defined range of activity that is the density (*y*-axis). The vertical lines are the means, with two means being shown for the bimodal plots (for the resistant and sensitive groups). **F,** Principal component analysis of GDSC *z*-score drug activity for Acetalax and bisacodyl as compared with the FDA-approved drugs for all (GDSC) cell lines. The drug names in purple are currently used for treatment of TNBC. The proportion of variance explained by PC2 is 6%. **G,** Principal component analysis of GDSC *z*-score drug activity for Acetalax and bisacodyl as compared with the clinical trial drugs for all (GDSC) cell lines. For both **F** and **G,** each circle is a drug. Both plots visualize PC1 (*x*-axis) vs. PC3 (*y*-axis). The proportion of variance explained by PC2 is 7.9%. **H,** Hierarchical clustering of the GDSC FDA-approved drug *z*-score data for the TNBC cell lines using 1-Pearson correlation distance (*x*-axis) and average linkage. The 58 drugs plus Acetalax and bisacodyl were included. The *y*-axis displays the drugs, with colors corresponding to the mechanisms of action (see legend). exp, expression; PCA, principal component analysis; PC, principal component.

Figure 1A and B visualizes the identification of the GDSC TNBC cell lines, defined by their low expression of *ESR1*, *PGR*, and *ERBB2*. Figure 1C shows that the FDA-approved oncology drugs (in gray) have a largely normal distribution with a mean of −0.25 (*z*-score). Drugs in clinical trial have a similar profile with a mean of −0.15 (*z*-score). Both Acetalax (Fig. 1D) and bisacodyl (Fig. 1E) have a clear bimodal response to the TNBC cell lines with ∼36% (eight of 22) of the responses clearly in the positive range for both drugs. The mean *z*-score activity of 2.4 for the more active (right) peak for both Acetalax and bisacodyl in triple negatives is substantially above the means of the onco-drugs, whereas the more resistant peak is of comparable activity with the oncology drugs. Pearson correlation comparison of the activities of Acetalax and bisacodyl demonstrates significance for both the TNBC (r = 0.97, *P* = 6.1E−14, *n* = 22) and all GDSC cancer cell lines (r = 0.92, *P* = 1.1E−297, *n* = 713). There is also a wide gap between the Pearson correlation of activity patterns of the *P* values of these two drugs as compared with all other GDSC drugs, with the *P* value of the next closest compound’s *P* value reduced by log_10_E−284 for Acetalax and log_10_E−280 for bisacodyl (for all cells). Principal component analysis makes a supportive point demonstrating the separation between the activity patterns of the Acetalax and bisacodyl as compared with either the 58 FDA-approved (Fig. 1F) or 58 clinical trial (Fig. 1G) drugs. The hierarchical clustering shown in Fig. 1H demonstrates that Acetalax and bisacodyl have greater separation from the other FDA-approved drugs than any of those drugs as compared with one another.

**Figure 2:**
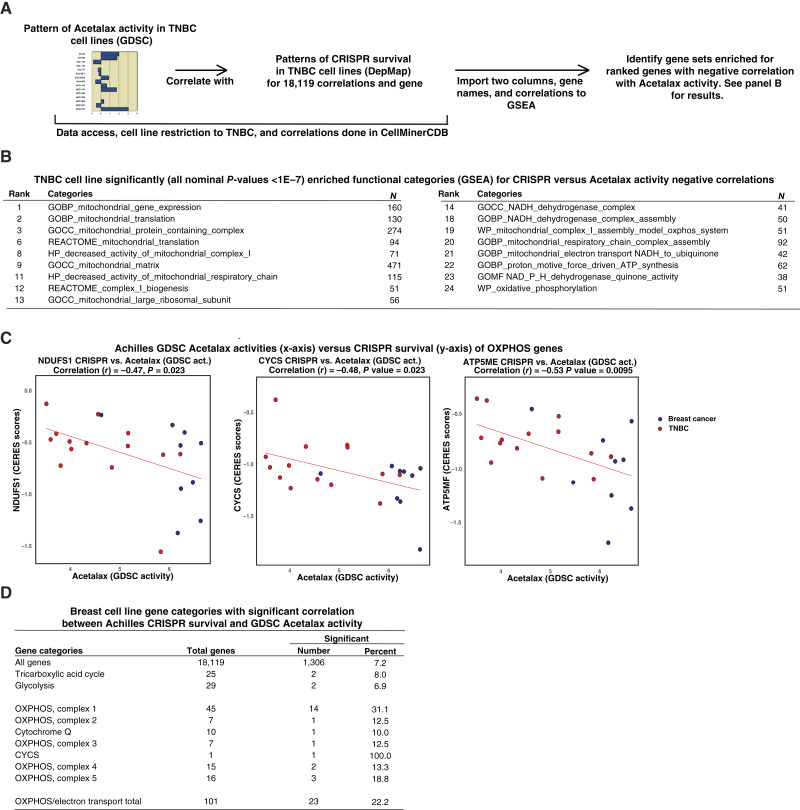
Implication of the mitochondrial pathways in cellular responses to Acetalax assessed by CRISPR (Achilles project, DepMap MIT) survival comparisons with Acetalax activity [GDSC_MGH-Sanger, −log_10_(IC_50_M)]. **A,** Schematic of the GSEA analysis workflow used to generate **B**. **B,** GSEA used to determine enriched categories of 18,121 genes from the comparison of CRISPR survival with Acetalax [−log_10_(IC_50_M)] activity for TNBC cell lines. *N* is the number of genes in the category. All nominal *P* values for these categories are less than 1 × 10^−7^. **C,** Scatter plots of Acetalax activity (*x*-axis) vs. CRISPR survival for three representative genes, *NDUFS1*, *CYCS*, and *ATP5ME* (*y*-axes) as specific examples from the **B** analysis. Each circle is a breast cell line, with the TNBC cell lines in red. The correlation type is Pearson. The red line is the regression line. Plots were generated using CellMinerCDB using Select Tissues\To include “Breast”. **D,** Levels of significant correlation in breast cancer cell lines for mitochondrial categories as compared with Acetalax activity in the breast cell line subset. The correlation between Acetalax activity and CRISPR gene survival data were generated from CellMinerCDB with the selection of “Breast” as the “To include” category. act, activity.

Figure 2A provides the workflow used in the generation of the Fig. 2B gene sets, which identifies pathways with significant relationships between Acetalax activity and CRISPR survival in TNBC. The functional categories enriched for significant negative correlation (all with nominal *P* values less than 1E−7) show a preponderance of mitochondrial/electron transport/OXPHOS categories, including 17 (68%) of the top 25 categories. Supplementary Table S1 lists the top 100 gene sets ranked by the nominal enrichment score in rank order and Fig. 2C provides three representative genes with significantly negatively correlated CRISPR survival versus Acetalax activity comparisons. The negative correlations indicate that the same cell lines most sensitive to the Acetalax are also the ones for which survival is more negatively affected by the CRISPR knockdowns of these genes. Figure 2D provides a more focused analysis for genes involved in the electron transport/OXPHOS processes shown to be enriched in Fig. 2B using the breast subset of cell lines. Together, they indicate that the genes for which CRISPR knockdown is significantly correlated with Acetalax activity are enriched in the electron transport/OXPHOS genes to a level of 22.2% as compared with the 7.2% overall level (for the 18,119 genes with CRISPR measurement). By the binomial test, this enrichment is significant with a *P* value of 8.9E−9. Supplementary Figure S1 presents the analogous panels A, B, and C showing results for bisacodyl activity as compared with CRISPR survival, with a similar emphasis seen in the mitochondrial/electron transport/OXPHOS categories. Supplementary Table S2 lists the top 100 gene sets in the rank order from Supplementary Fig. S1B.

### EMT analyses

Supplementary Figure S2A shows the schema used to determine the genes and pathways associated with Acetalax response in the TNBC cell lines, and Supplementary Fig. S2B shows GSEA in the Acetalax-sensitive (left) and bisacodyl-sensitive (right) cell lines. For both drugs, transcript expressions were compared for five most sensitive versus five most resistant cell lines. EMT-related gene sets were obtained for 10 of the top 25 (40%) categories enriched in the sensitive cell lines for both the Acetalax and bisacodyl analyses. The gene sets for the sensitive cell lines indicate higher epithelial (luminal) characteristic whereas the resistant indicate higher mesenchymal (basal) characteristic for both drugs. In Supplementary Fig. S2C, the first two scatter plots from the left provide an example of a representative epithelial gene, EPN3, with significant correlation with Acetalax activity for both the breast subset and all cell lines. The right two panels demonstrate that cells rated as epithelial with positive EMT scores also have significant positive correlation to Acetalax activity. The two EMT plots shown on the right panels of Supplementary Fig. S2C confirm that cells with increased epithelial characteristic tend to have higher sensitivity to Acetalax, whereas the more mesenchymal cells (with negative EMT scores) are more resistant. To assess the relationship between EMT status and other drugs, we broadened the EMT signature comparisons with the other FDA-approved drugs for the TNBC subgroup and found four other drugs that were significantly more active in the epithelial cells, which were belinostat, 5-fluorouracil, methotrexate, and gefitinib. The drugs significantly more active in the mesenchymal cells were cytarabine and palbociclib.

### *In vivo* antitumor activity of Acetalax and bisacodyl in PDX models of TNBC

**Figure 3:**
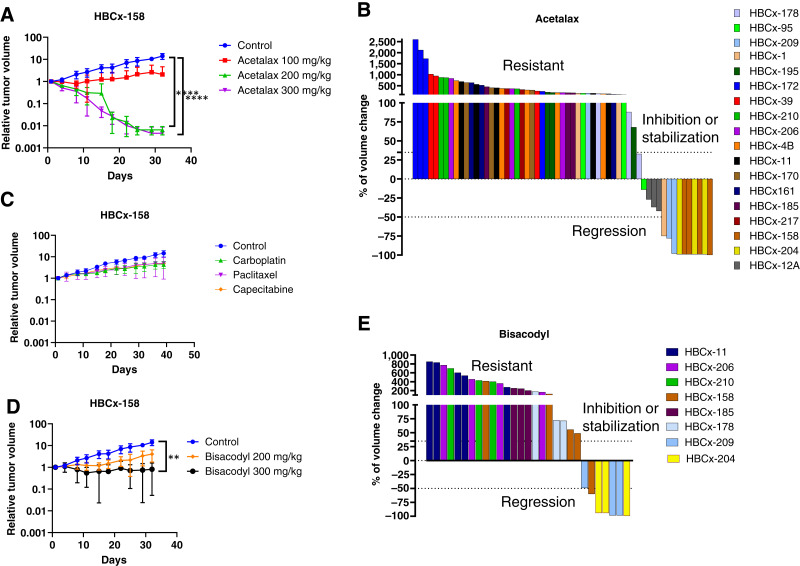
*In vivo* response to Acetalax and bisacodyl in PDX models of TNBC. **A,***In vivo* antitumor effect of Acetalax administered at 100, 200, and 300 mg/kg in the HBCx-158 PDX model. Mean ± standard deviation (*n* = 4–6 mice/group). ****, *P* < 0.0001 (Kruskal–Wallis test). **B,** Waterfall plot showing the percentage of tumor volume changes in xenografts treated with Acetalax (200 mg/kg) in 18 different PDX models. Each bar represents a single mouse. **C,***In vivo* response of HBCx-158 to carboplatin (90 mg/kg), paclitaxel (25 mg/kg), and capecitabine (540 mg/kg) chemotherapies. **D,***In vivo* antitumor activity of bisacodyl administered at 200 and 300 mg/kg in the HBCx-158 PDX model. Mean ± standard deviation (*n* = 4–6 mice/group). **E,** Waterfall plot showing the percentage of tumor volume changes in xenografts treated by bisacodyl (200 mg/kg) from eight different PDX models. Each bar represents a single mouse. For both **B** and **E**, the responses are grouped into three categories, with the top being resistant, the middle inhibition or stabilization, and the bottom regression. For **A**, **C**, and **D**, the *y*-axis units are log_10_.

Given the sensitivity of TNBC cell lines to Acetalax, its antitumor activity was evaluated *in vivo* in 18 PDX models of early-stage TNBC established from treatment-naïve breast tumors (*n* = 4) or residual tumors after neoadjuvant chemotherapy (*n* = 14). The histologic characteristics and the mutational profiles of the 18 PDXs are summarized in Table 1. We first performed a dose–response study in the HBCx-158 PDX (Fig. 3A) by testing 100, 200, and 300 mg/kg doses administered 5 days/week for 5 weeks. Responses to Acetalax and bisacodyl were defined from the percentage of tumor volume change calculated at the end of the experiment. Treatment with Acetalax at 200 and 300 mg/kg doses led to statistically significant change, with the tumor volume going to zero, whereas the 100 mg/kg dose resulted in SD. The 200 mg/kg dose was chosen to test the additional 17 models of TNBC (between two and five mice were tested for each PDX model), and the results are summarized in Fig. 3B as a waterfall plot, with each column being an individual mouse. Of the 18 PDX models, two responded with complete response (HBCx-204 and HBCx-158), one with partial response (HBCx-209), one with SD (HBCx-12A), and the remaining did not respond (PD; Table 1; Fig. 3B).

**Table 1:** List of PDXs tested for Acetalax and bisacodyl responses

PDX	PDX origin	Histology	Mutation	Acetalax response	Bisacodyl response
HBCx-152	Residual tumor	IBC-NST	*TP53*	**CR**	SD at 300 PD at 200
HBCx-39	Residual tumor	IBC-NST	*TP53*	PD	
HBCx-1	Primary tumor	IBC-NST		PD	
HBCx-11	Primary tumor	IBC-NST	*BRCA1*	PD	PD
HBCx-4B	Primary tumor	IBC-NST	*PIK3CA*	PD	
HBCx-195	Residual tumor	IBC-NST	*TP53*	PD	
HBCx-95	Residual tumor	IBC-NST		PD	
HBCx-12A	Residual tumor	IBC-NST	*NOTCH4*	**SD**	
HBCx-161	Primary tumor	IBC-NST	*TP53*	PD	
HBCx-170	Residual tumor	IBC-NST	*TP53*	PD	
HBCx-185	Residual tumor	Metaplastic BC	*TP53*	PD	PD
HBCx-210	Residual tumor	IBC-NST	*TP53*	PD	PD
HBCx-178	Residual tumor	Metaplastic BC	*TP53* and *PIK3CA*	PD	PD
HBCx-206	Residual tumor	IBC-NST	*TP53*, *RB1*, and *RAD54L*	PD	PD
HBCx-209	Residual tumor	IBC-NST	*BRCA1* and *TP53*	**PR**	**CR**
HBCx-217	Residual tumor	IBC-NST	*TP53*	PD	
HBCx-174	Residual tumor	IBC-NST	*TP53* and *FATA4*	PD	
HBCx-204	Residual tumor	IBC-NST	*TP53* and *NOTCH1*	**CR**	**CR**

Abbreviations: BC, breast cancer; CR, complete response; HBCx, human breast cancer xenograft; IBC-NST, invasive carcinoma of no special type; PR, partial response.

Importantly, treatment by Acetalax resulted in antitumor activity in a subset of TNBC PDXs established from patients treated in the neoadjuvant setting and showing residual disease at surgery, with a high risk of tumor recurrence and a particularly poor prognosis. Moreover, some of the most sensitive PDXs to Acetalax or bisacodyl show resistance to standard chemotherapies used in breast cancer. The HBCx-12A PDX, which showed tumor mass reduction, is cross-resistant to AC (Adriamycin + cyclophosphamide), cisplatin, and docetaxel (24). HBCx-158, which had complete tumor elimination by Acetalax, was cross-resistant to paclitaxel, carboplatin, and capecitabine (Fig. 3C).

We also tested the antitumor activity of bisacodyl which is still used worldwide as a laxative in eight of the 18 PDXs. Bisacodyl treatment resulted in SD when dosed at 300 mg/kg and PD when administered at 200 mg/kg (Fig. 3D) for HBCx158. PDXs HBCx-209 and HBCx-204 responded to bisacodyl with complete response (Supplementary Fig. S3). The other five models were found to be resistant to bisacodyl treatment (Fig. 3E). These results identify a subset of TNBC as sensitive to bisacodyl. Additional statistical analysis showing significance of efficacy for both Acetalax and bisacodyl for PDXs HBCx-204 and HBCx-209 and of Acetalax for HBCx-12A is supplied in Supplementary Fig. S3. It is to be presumed that working at 300 mg/kg would have led to improved results for the PDXs described in both Fig. 3D and E.

### *In vivo* pharmacodynamic analysis of Acetalax and bisacodyl

**Figure 4:**
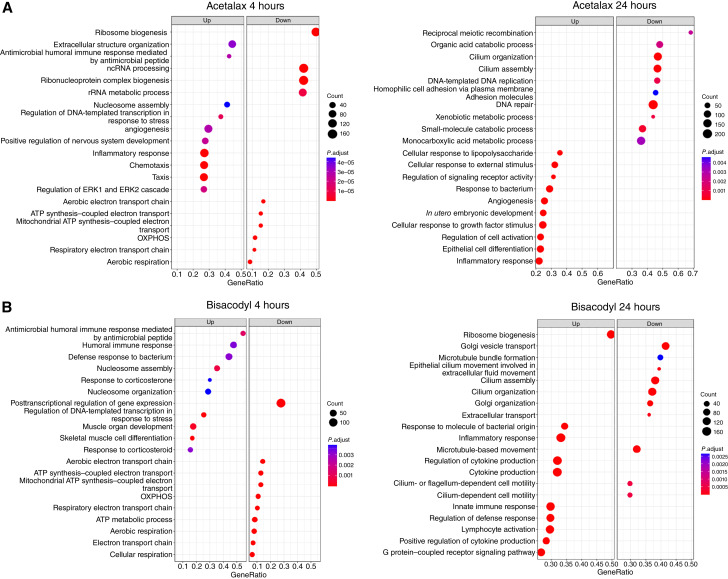
GSEA of RNA-seq for PDX HBCx-185 PDX treated with Acetalax or bisacodyl. **A,** Acetalax treatment at 4 hours (left plot) and 24 hours (right plot) as compared with no drug controls. **B,** Bisacodyl treatment at 4 hours (left plot) and 24 hours (right plot) as compared with no drug controls. For all experiments, four to five mice were used as biological replicates for both the drug treated and no drug controls. All categories shown have significant enrichment (*P* < 0.0008).

To identify the biological processes altered in response to Acetalax and their potential mechanisms of actions, a pharmacodynamic study was performed in the Acetalax-responder PDX HBCx-158. HBCx-158 xenografts were treated with a single dose of Acetalax and tumors were analyzed by RNA-seq at 4 and 24 hours after treatment. Results of GSEA are provided in Supplementary Tables S3–S6. The top functions activated or suppressed in response to Acetalax are shown in Fig. 4A. Acetalax suppresses ribosome biogenesis, ncRNA processing, and multiple mitochondrial/respiratory/ATP synthesis categories at 4 hours (left). At 24 hours after treatment (right), Acetalax inhibited cilium organization and assembly and suppressed DNA repair and carboxylic acid catabolic processes.

To identify the biological processes altered in response to bisacodyl, the same design as Acetalax was used. The top functions activated or suppressed in response to bisacodyl are shown in Fig. 4B. At 4 hours, tumors treated with bisacodyl showed inhibition of OXPHOS, aerobic respiration, mitochondrial ATP synthesis, and energy production, overlapping with the Fig. 4A Acetalax response (left). At 24 hours after treatment, cilium organization and assembly were inhibited (right).

**Figure 5:**
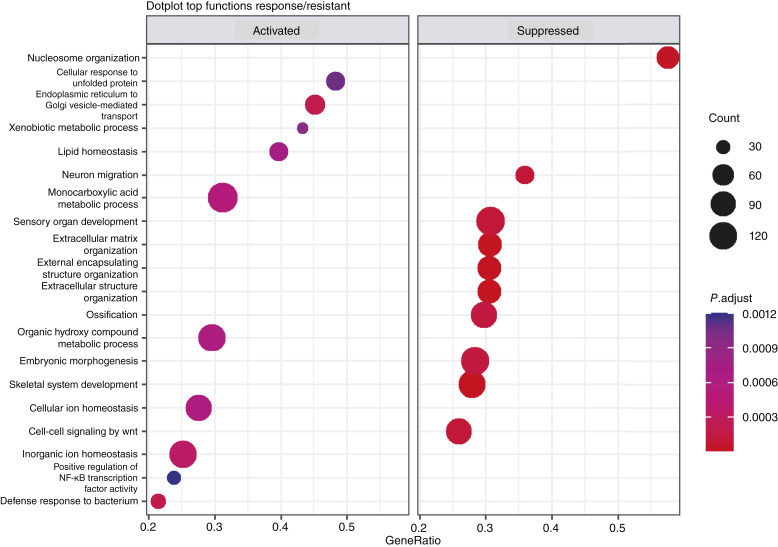
GSEA of RNA-seq data of four Acetalax-sensitive PDX tumors vs. eight resistant, as compared with no drug controls. The Acetalax-sensitive PDXs were HBCx-209, HBCx-HBCx-12A, HBCx-204, and HBCx-158. The Acetalax-resistant PDXs were HBCx-39, HBCx-11, HBCx-4, HBCx-170, HBCx-185, HBCx-210, HBCx-217, and HBCx-172.

To identify potential mechanisms or pathways driving response or resistance to Acetalax, we performed a second analysis of the RNA-seq comparing the four responder PDXs (HBCx-209, HBCx-HBCx-12A, HBCx-204, and HBCx-158) to eight resistant PDXs (HBCx-39, HBCx-11, HBCx-4, HBCx-170, HBCx-185, HBCx-210, HBCx-217, and HBCx-172) at baseline levels (untreated tumors). The results of the subsequent GSEA analysis are summarized in Fig. 5 and detailed in Supplementary Table S7. The Fig. 5 (left) dot plot representing the top functions for the Acetalax-responder tumors shows upregulation of gene sets associated with ion homeostasis, fatty acid metabolism, and response to viruses. Conversely, gene sets such as regulation of DNA recombination and protein–DNA complex subunit organization are down-regulated in the Acetalax-responsive tumors.

### Exploration of transcripts predictive of Acetalax activity

**Figure 6:**
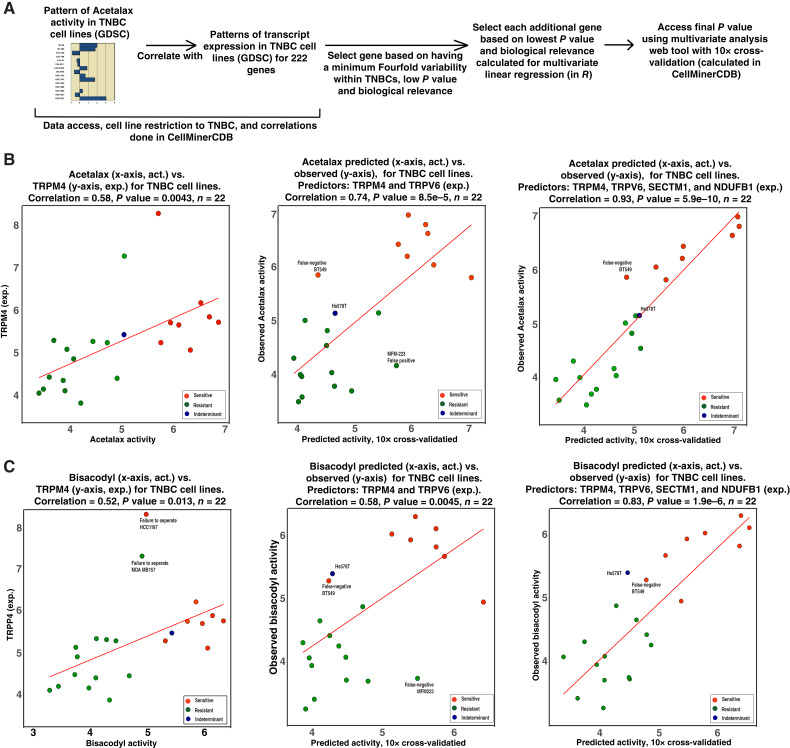
Prediction of Acetalax and bisacodyl activity based on transcript expression in the TNBC cell lines of the GDSC. **A,** Schematic of the approach used to generate **B** and **C**. **B,** Prediction of Acetalax activity based on transcript expression. The left panel is correlation. The middle and right panels demonstrate increasing levels of prediction when including additional genes in multivariate analysis (calculated in R). **C,** Same as **B** for bisacodyl. For *P* value determination of the multigene panels, we used our multivariate analysis web tool (https://discover.nci.nih.gov/rsconnect/cellminercdb/) with 10× cross-validation. The drug activity and transcript (microarray) levels referred to in all panels were as measured by GDSC. The circles are cell lines. The red lines are regression lines. act, activity; exp, expression.

Using the approach summarized in Fig. 6A and CellMinerCDB (https://discover.nci.nih.gov/; ref. 11), we showed that *TRPM4* gene expression has significant correlation with both Acetalax and bisacodyl activity in the TNBC cell lines of the GDSC (Fig. 6B and C, left scatter plot for both).

By increasing the number of genes and switching to multivariate analysis, one may explore the use of multiple genes to more accurately predict the activity of a given drug (11, 25). Figure 6B shows that the two and four gene predictions (center and right scatter plots) yielded improved Acetalax activity prediction. Panel C provides the analogous analysis for bisacodyl with similar results. Supplementary Figure S4A–S4C provides the Acetalax activity versus single-gene transcript pattern correlations and significance for *TRPV6*, *SECTM1*, and *NDUFB1* (all used in the Fig. 6B analyses).

We next extended these results to the *in vivo* work with comparison of tumor size (Fig. 3) and transcript expression as affected by drugs for the PDXs (Figs. 4 and 5; Supplementary Fig. S5). Of the four genes found to predict both Acetalax and bisacodyl activity in the TNBC cell lines (Fig. 6), one (*TRPM4*, *P* = 0.0091) was found to have significant correlation with tumor size for both Acetalax and bisacodyl (Supplementary Fig. S5A–S5C) using the three categories from Fig. 3B and E (regression, inhibition, or stabilization and resistance). The Acetalax comparison using the tumor size as continuous values (Supplementary Fig. S5B) found significant correlation for *NDUFB1* (*P* = 0.038). The bisacodyl discreet approach using discreet values (Supplementary Fig. S5C) found significant correlation for *SECTM1* (*P* = 0.0013). Comparison of differential expression in responder versus resistant PDXs (Fig. 5) also yielded significant correlation in the Fig. 6 cell line analysis genes; that is, the two results mirrored one another. The Fig. 5 is showing transcript expression versus Fig. 3 is showing tumor size correlation for the *TRPM4* (*P* = 0.00063), *SECTM1* (*P* = 0.024), *TRPV6* (*P* = 0.029), and *NDUFB1* (*P* = 0.014) in the absence of multiple comparisons correction.

### Pharmacokinetics of Acetalax

**Figure 7:**
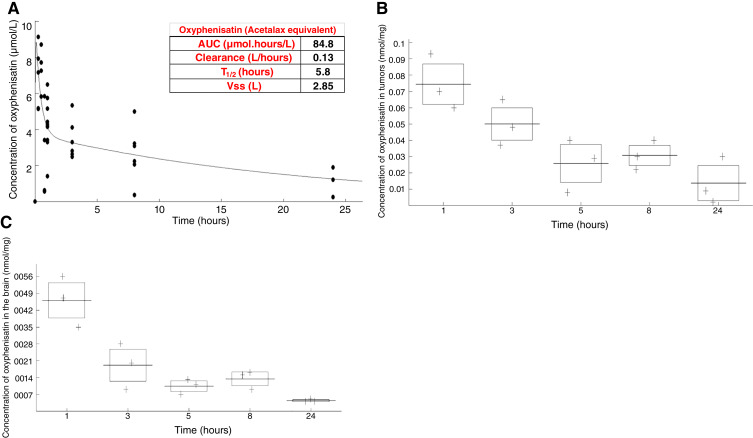
Pharmacokinetics of Acetalax in plasma, tumor and several organs from PDX HBCx-158. **A,** Plasma concentrations of oxyphenisatin vs. time curve. **B,** Concentrations of oxyphenisatin in tumor vs. time. **C,** Concentrations of oxyphenisatin in brain vs. time. The squares depict one standard deviation for the results and the line the mean.

To characterize the pharmacokinetic properties of Acetalax, a study was performed in the highly responsive PDX (HBCx-158). Plasma, organs, and tumor were analyzed at different times after a single dose of Acetalax. Due to a rapid metabolism of Acetalax into oxyphenisatin by esterases in mice, we hypothesized that 1 mol of Acetalax is transformed into 1 mol of oxyphenisatin (with acetate removed). The pharmacokinetic parameters of oxyphenisatin are summarized in Fig. 7A. Plasma concentrations of oxyphenisatin declined biexponentially after dosing, and oxyphenisatin concentrations in plasma remained above the limit of quantification until 24 hours after dose for all animals. Mean half-life was about 5.8 hours. Over the 24-hour period following the day 1 administration, 1% and 0.1% of the dose was recovered in the tumor and brain, respectively (Fig. 7B and C). Over the same 24-hour period following the day 1 administration, 4.5% and 72% of the dose was recovered in the kidney and liver, respectively. These results show that Acetalax is distributed to tumor, crosses the blood–brain barrier, and that its blood pharmacokinetics is acceptable for further studies.

## Discussion

With the relatively poor prognosis of TNBC, more effective treatments for the disease are warranted. To that end, we have identified from the GDSC breast cancer cohort 25 TNBC cell lines as a drug-assessing model (Fig. 1A and B). Our approach (Fig. 1C–E) provides an informative comparison of existing approved oncology drugs with Acetalax and bisacodyl utilizing *z*-scores taken across all cell lines. The data in this form identify the TNBC’s relative sensitivity when compared with the other FDA-approved and clinical trial drugs included in the screen. The distribution of the oncology drug responses indicates only sporadic positive activities, similar to the clinical experience. In contrast, the non-oncology Acetalax and bisacodyl have preferential activity in subsets of TNBC as reflected by bimodal splits (Fig. 1D and E). Of note, the more resistant responses decrease in the same range as the currently used oncology drugs. The positive responders are identifying a subpopulation of TNBCs susceptible to Acetalax and bisacodyl in contrast to approved oncology drugs. In fact, Acetalax and bisacodyl outperform gemcitabine, doxorubicin, cisplatin, olaparib, talazoparib, paclitaxel, and docetaxel, all drugs used clinically in the treatment of TNBC (26).

Comparison of the activity patterns of Acetalax and bisacodyl reveals their similarities both in TNBC cell lines and PDXs. This implies a comparable mechanism of action for the two drugs, with Acetalax being somewhat more active, consistent with a parallel study in selected TNBC cell lines (12). In addition, the wide gap of correlation *P* values between Acetalax and bisacodyl and between these and the next closest compounds suggests the unique mechanism of action of Acetalax and bisacodyl, likely involving oncosis and inflammatory cell death as opposed to apoptosis (12). In a parallel study, we discovered a unified mechanism of activity for both Acetalax and bisacodyl in cancer cells that likely involves the trapping of TRPM4 followed by its rapid degradation, cell membrane disruption, increased cellular volume, and mitochondrial defects with ATP depletion (12).

A relationship between mitochondrial dysfunction and Acetalax activity is implied by the preponderance of genes in the mitochondrial/electron transport/OXPHOS categories in GSEA of Acetalax activity versus CRISPR knockdown in cell lines (see Fig. 2). Analysis of this type implies the involvement of multiple genes from these categories (Fig. 2B), a point supported by the enriched number of genes with significant correlation with Acetalax activity in the mitochondrial genes (Fig. 2D). These results are consistent with multiple prior indicators from related studies of mitochondrial association with the cellular response to Acetalax. These include Acetalax treatment being associated with (i) increased anaerobic glycolysis by inhibition of respiration, (ii) decrease in the ATP:AMP ratio, (iii) activation of the ATP sensor PRKAA1, (iv) a 43-fold reduction of Acetalax activity in drug combination assays with AICAR, a PRKAA1 (alias AMPK) ATP sensor activator, (v) rapid downregulation of the mitochondrial membrane anti-apoptotic protein BCL2, (vi) a shift in the mitochondrial location to perinuclear, and (vii) mitochondrial membrane depolarization and (viii) reduced mitochondrial membrane potential in response to Acetalax (9, 12, 27). Consistent with the importance of mitochondrial dysfunction in response to Acetalax and bisacodyl, the second most correlated drug with both Acetalax and bisacodyl using a drug activity pattern comparison analysis is dihydrorotenone, a mitochondrial inhibitor (28). Additionally, TNBC stem cells have been proposed to be inhibited or killed by the mitochondrion-affecting drugs pyrvinium pamoate and ONC201, providing a prospective conceptual link (5, 29).

The Kohn EMT epithelial category (refs. 11, 30; rank 7) referred to in the Supplementary Fig. S2B and used on the *y*-axis in the Supplementary Fig. S2C (two right panels) is a 38-transcript signature described previously (6, 30), which we also found to be associated with Acetalax and bisacodyl activity (6). EMT drives enhanced invasiveness and metastatic properties in addition to the reported generation of chemoresistant breast cancer stem cells (31), and therefore, Acetalax and bisacodyl may prove useful under these conditions.

Using a subset of five TNBC cell lines in the GDSC, we recently identified that a key target of Acetalax and bisacodyl is the plasma membrane protein TRPM4, which also has significant correlation to the full set of GDSC cell lines (12). Our results from Fig. 6B and C extends this to the TNBC subset. The expression of TRPM4 is significantly related to the activity of Acetalax and bisacodyl. Hs578T in the Fig. 6B and C scatter plots (left) is depicted as indeterminate with regard to being sensitive or resistant, as it falls on both sides of an arbitrary Acetalax activity cutoff of 5.2, being somewhat more resistant for Acetalax (5.17) and more sensitive for bisacodyl (5.42). TRPM4 is overexpressed in several forms of cancer, and therefore could be considered a prospective prognostic marker and a promising anticancer target (32). *TRPV6*, a second gene associated with ion fluxes at the plasma membrane was the second most correlated gene with Acetalax activity in the CellMinerCDB (https://discover.nci.nih.gov/). It is a prospective regulator of TRPM4 and also considered to be a promising drug target for breast cancer (33). *NDUFB1*, a mitochondrial OXPHOS complex 1 gene adds to the predictive model for Acetalax activity (see Fig. 6B) and is consistent with the Fig. 2B and D results and a prior report of ATP depletion resulting from either Acetalax or bisacodyl treatment (11). The biological advantage of multivariate analysis is that it allows one to begin to explore the interactions of the multiple genes involved in a pharmacologic response. NDUFB1 provides an illustration of this type of exploration in that although it does not by itself have significant correlation to Acetalax (Supplementary Fig. S4C), it adds to the predictive power of the Fig. 6B multigene model in addition to having biological linkage to the ATP levels of sensitive versus resistant cell lines to these drugs (12).

With regard to any relationship between *TRPM4* expression and drugs currently used to treat TNBC, we checked the GDSC cell lines and there was no significant correlation with 5-fluorouracil, cisplatin, dabrafenib, docetaxel, doxorubicin, gemcitabine, olaparib, paclitaxel, and topotecan. Expression of prospective biomarkers for heterogeneity within TNBC were compared with Acetalax activity yielding significant correlations to CRTC2 for multiple drugs in the PI3K/AKT/mTOR-activated category as well as IL12B and LAG3 for multiple drugs within the immune-enriched category (34).

That *TRPM4* and *NDUFB1* were both found to be predictive of both Acetalax and bisacodyl activity in cell lines (Fig. 6) and were also found to significantly correlate with PDX tumor response to Acetalax (Fig. 3; Supplementary Fig. S5) and have differential expression in responders versus resistant PDXs (Fig. 5) indicates functional concordance between the cell line and PDX models. That *TRPM4* was the sole gene to survive multiple comparison correction *in vivo* (Supplementary Fig. S5) highlights this gene’s prospective importance to the drug response, consistent with prior results (11). The relevance of TRPM4 as a drug target and biomarker for Acetalax and bisacodyl, which we discovered in a parallel study (12), highlights the potential of TRPM4 for developing additional drugs targeting the plasma membrane of cancer cells. *TRPM4* expression from The Cancer Genome Atlas breast cancer tissues was found to have significant correlation with patient survival (*P* = 0.004, from OncoLnc, http://www.oncolnc.org/). A recent study from the Shapiro group is consistent with this possibility as TRPM4 is the target of two related compounds, ErSO and BHP1, which like Acetalax and bisacodyl exhibit antitumor activity in murine models expressing TRPM4 (15). Notably, the activity of ErSO (NSC 835589) is highly correlated with the activity of Acetalax in the NCI60 (Pearson correlation r = 0.84; *P* value = 1.8E−16, Supplementary Fig. S6A) and with the expression of *TRMP4* (Pearson correlation r = 0.5; *P* value = 7.6E−05, Supplementary Fig. S6B). Together, these results suggest TRPM4 as a novel target for drug development.

The pharmacokinetics results suggest that Acetalax penetrates tumors, crosses the blood–brain barrier, and has an appropriate blood half-life of 5 to 6 hours. Of note, the patients used to generate the PDXs (Fig. 3) in this study have undergone neo-adjuvant treatment and have residual disease at surgery and have positive responses. This is the same group of patients likely to be used if the drugs are brought to clinical trial. The Fig. 7A pharmacokinetics provides a measure of the amount of Acetalax in the plasma. Using this and back calculating to the average amount of plasma in humans, a recommended dose of 9 mg of Acetalax is derived (in humans), an amount that should be easily achievable with intravenous administration. Another prospective clinical consideration comes from the following. Most TNBC deaths come from distant metastasis (35). Brain metastasis occurs in some 30% of patients with TNBC (36). Systemic chemotherapy is ineffective in treating these brain metastasis largely because of the blood–brain barrier (37). Currently, there is no standard treatment for TNBC brain metastasis (38). As their occurrence is an exclusion criterion for clinical trials, this serves as an impediment to progress for this patient population (36). The ability of Acetalax to penetrate the blood–brain barrier demonstrated in Fig. 7C provides both an advantage over standard chemotherapies and a rationale for further investigation of this drug for this patient population.

In anticipation of a prospective move toward clinical application for these drugs, several points will need to be kept in mind. First, the drugs will need to be reformulated for intravenous injection. Second, those reformulated drugs will require formal toxicology. Third, the drugs will then require phase I assessment to monitor and assess any toxicities such as liver enzyme elevation as well as determination of the recommended phase II dosage.

## Supplementary Material

Supplementary Figure 1 CRISPR (Achilles project) survival comparisons to bisacodyl activity.

Supplementary Figure 2 Comparisons of acetalax activity to transcript level and epithelial mesenchymal transition (EMT) score.

Supplementary Figure 3 In vivo response to Acetalax and/or bisacodyl in patient derived xenograph (PDX) models of TNBC.

Supplementary Figure 4 Scatter plots of gene transcript levels versus acetalax activity for genes from the Figure 6 multivariate analysis.

Supplementary Figure 5 Tumor mass response as compared to untreated transcript expression.

Supplementary Figure 6 Scatter plots of ErSO drug activity versus acetalax activity and TRPM4 gene transcript levels.

Supplementary Table 1 Supplementary Table 1

Supplementary Table 2 Supplementary Table 2

Supplementary Table 3 Supplementary Table 3

Supplementary Table 4 Supplementary Table 4

Supplementary Table 5 Supplementary Table 5

Supplementary Table 6 Supplementary Table 6

Supplementary Table 7 Supplementary Table 7
