# Supplementary material for: Acetalax and Bisacodyl for the Treatment of Triple-Negative Breast Cancer: A Combined Molecular and Preclinical Study
Source: Cancer Res Commun. 2025 Feb 28;5(2):375–88. doi: 10.1158/2767-9764.CRC-24-0435 (PMC11869203; doi:10.1158/2767-9764.CRC-24-0435)
Supplement: Supplementary Figure 1 — CRISPR (Achilles project) survival comparisons to bisacodyl activity. [file crc-24-0435_supplementary_figure_1_suppsf1.pdf]

Supplemental Figure 1

A.

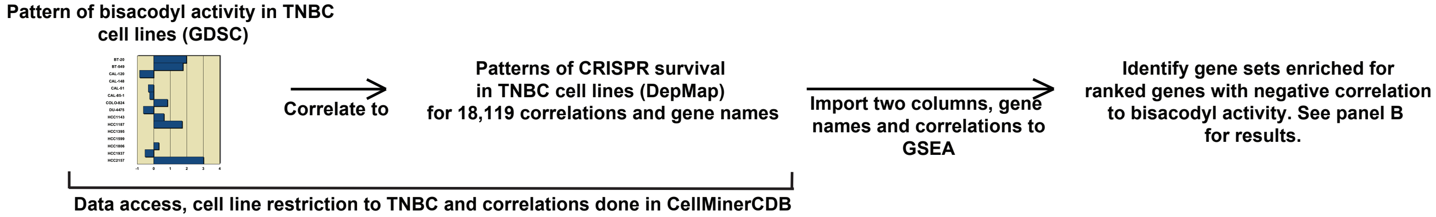

B.

TNBC cell line enriched functional categories for CRISPR survival genes significantly negatively correlated to bisacodyl activity

Rank	Categories	N
1	GOBP_Mitochondrial_gene_expression	160
2	GOBP_Mitochondrial_translation	130
6	GOCC_Mitochondrial_protein_containing_complex	274
9	REACTOME_Mitochondrial_translation	94
15	GOCC_Mitochondrial_large_ribosomal_subunit	56
16	HP_Decreased_activity_of_mitochondrial_respiratory_chain	115
17	GOCC_Mitochondrial_matrix	471
18	WP_Mitochondrial_complex_I_assembly_model_oxphos_system	50
20	GOMF_Oxidoreductase_activity_acting_on_NAD_P_H_quinone_or_similar...	50
21	GOCC_NADH_dehydrogenase_complex	41
22	GOBP_Mitochondrial_respiratory_chain_complex_assembly	92
23	WP_Oxidative_phosphorylation	51
24	GOBP_Mitochondrial_electron transport NADH_to_ubiquinone	42

C.

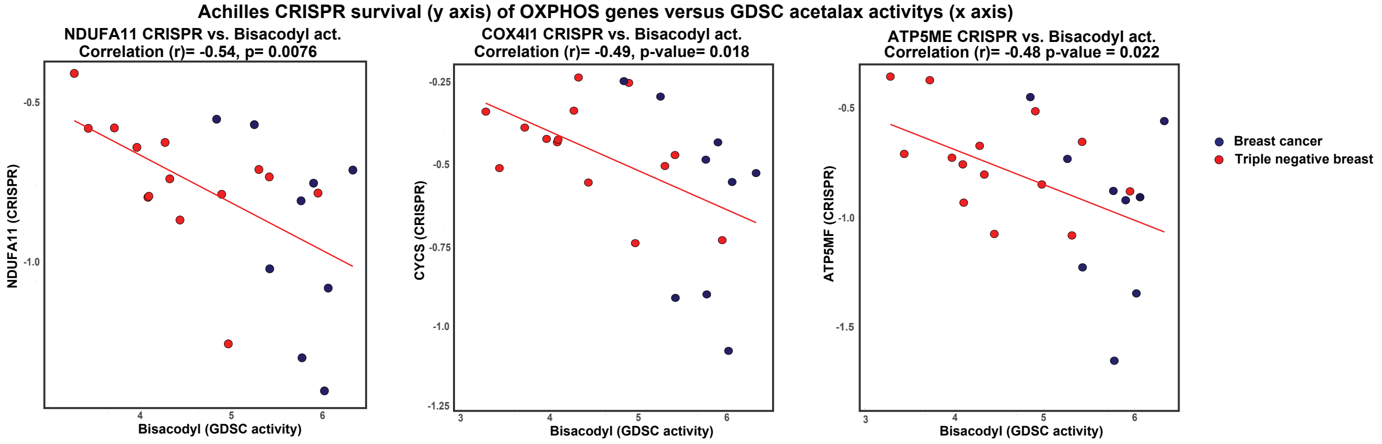

Legend. CRISPR (Achilles project) survival comparisons to bisacodyl activity (GDSC_MGH-Sanger, -log10(IC50M).
A. Schematic of GSEA analysis workflow presented in panel B. **B.** Gene Set Enrichment Analysis (GSEA) used to determine enriched categories of 18,121 genes from the comparison of CRISPR survival to bisacodyl activity for TNBC cell lines. N is the number of genes in the category. All nominal p-values for these categories are less than 1x10-7. **C.** Scatter plots of acetalax activity (x- axis) verses CRISPR survival of three representative genes NDUFA11, COX4I1 and ATP5ME (y axes), to provide specific examples from the panel B analysis. Each circle is a breast cell line, with the red triple negatives. The correlation type is Pearson’s. The red line is the regression line.
