# Supplementary material for: Acetalax and Bisacodyl for the Treatment of Triple-Negative Breast Cancer: A Combined Molecular and Preclinical Study
Source: Cancer Res Commun. 2025 Feb 28;5(2):375–88. doi: 10.1158/2767-9764.CRC-24-0435 (PMC11869203; doi:10.1158/2767-9764.CRC-24-0435)
Supplement: Supplementary Figure 2 — Comparisons of acetalax activity to transcript level and epithelial mesenchymal transition (EMT) score. [file crc-24-0435_supplementary_figure_2_suppsf2.pdf]

Supplemental Figure 2

A.

Pattern of acetalax activity in TNBC cell lines (GDSC)

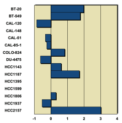

→ Correlate to → **Patterns of 19,652 gene's transcript expression in TNBC cell lines (GDSC)** → **Rank the correlations. from high to low** → **Import to GSEA** → **Identify gene sets enriched for ranked genes with correlation to acetalax or bisacodyl activity by comparison of 5 sensitive vs 5 resistant cell lines. See panel B for results.**

Data access, cell line restriction to TNBC and correlations done in CellMinerCDB

B.

Functional categories of TNBC gene transcripts significantly enriched in acetalax and bisacodyl sensitive cell lines

Categories enriched in the acetalax sensitive set

Rank	Categories	N
1	Charafe breast cancer luminal vs mesenchymal_up	420
2	Charafe breast cancer basal vs mesenchymal_up	112
4	Hollern EMT breast tumor_dn	118
6	Aigner ZEB1 targets	33
7	Kohn EMT epithelial	22
9	Onder CDH1 targets 2_dn	451
14	Foroutan TGFB EMT_dn	71
15	Foroutan integrated TGFB EMT_dn	101
16	Blanco melo beta interferon treated bronchial epithelial cells_dn	190
24	Bosco epithelial differentiation module	62

Categories enriched in the bisacodyl sensitive set

Rank	Categories	N
1	Charafe breast cancer basal vs mesenchymal_up	112
2	Hollern EMT breast tumor_dn	118
4	Charafe breast cancer luminal vs mesenchymal_up	420
7	Kohn EMT epithelial	22
8	Bosco epithelial differentiation module	62
10	Foroutan TGFB EMT_dn	101
11	Onder CDH1 targets 2_dn	451
13	Blanco melo beta interferon treated bronchial epithelial cells_dn	190
14	Foroutan integrated TGFB EMT_dn	71
15	Aigner ZEB1 targets	33

C.

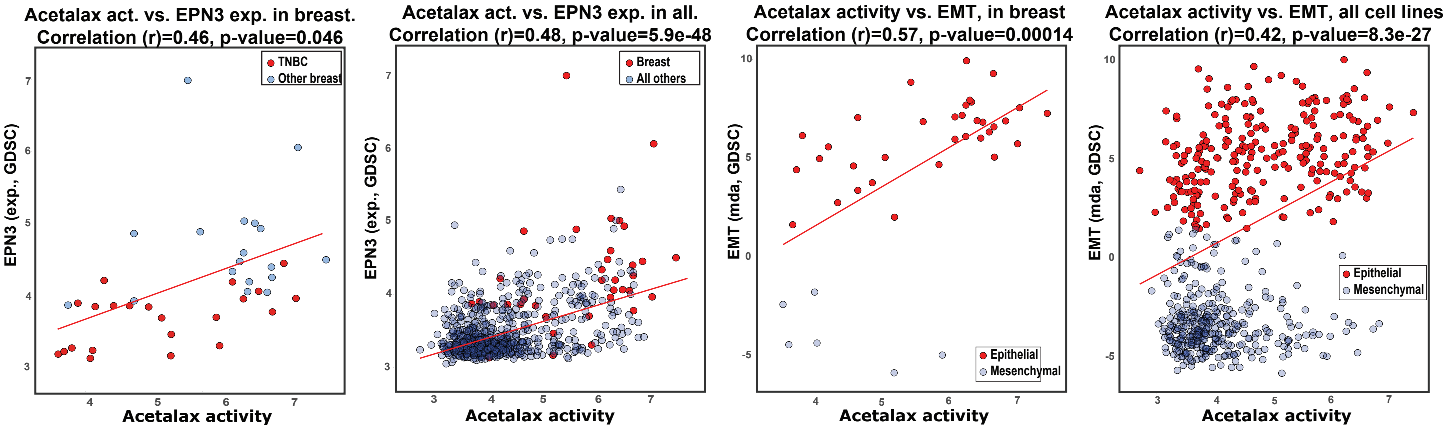

Legend: Comparisons of acetalax activity to transcript level and epithelial mesenchymal transition (EMT) score.

A. Schematic of the workflow used to generate the panel B results. **B.** GSEA comparison of transcript levels of five acetalax-sensitive versus five acetalax-resistant TNBC cell lines (left panel). GSEA comparison of transcript levels of five bisacodyl-sensitive versus five bisacodyl-resistant TNBC cell lines (right panel). The enriched category determination is done using the signal2noise metric and weighted scoring schema provided in the GSEA website. Ranking is by the nominal enrichment score. N is the number of genes in the category. All categories shown have significant enrichment ($p<0.0008$). **C.** Scatter plots of acetalax activity (x-axis) versus the EPN3 transcript expression (panels one and two) and the epithelial-mesenchymal transition (panels three and four) score (y-axes). The first and third panels are breast and the second and forth panels are all cancer cell lines. Each circle is a cell line, with the colors defined in the legends. The red line is a regression line. Mda is miscellaneous data, Act is activity, EMT is epithelial mesenchymal transition, and NSC is national service center number. For all panels the drug activities are as measured by GDSC (-log10(IC50M)). Scatter plots were generated using CellMinerCDB. For the first and the third plots "Select Tissues \ To include, Breast" was used. For the second and the fourth plots, "all cell lines" were included.
