# Supplementary material for: Acetalax and Bisacodyl for the Treatment of Triple-Negative Breast Cancer: A Combined Molecular and Preclinical Study
Source: Cancer Res Commun. 2025 Feb 28;5(2):375–88. doi: 10.1158/2767-9764.CRC-24-0435 (PMC11869203; doi:10.1158/2767-9764.CRC-24-0435)
Supplement: Supplementary Figure 3 — In vivo response to Acetalax and/or bisacodyl in patient derived xenograph (PDX) models of TNBC. [file crc-24-0435_supplementary_figure_3_suppsf3.pdf]

Supplemental Figure 3

HBCx-204 (individual xenografts curves)

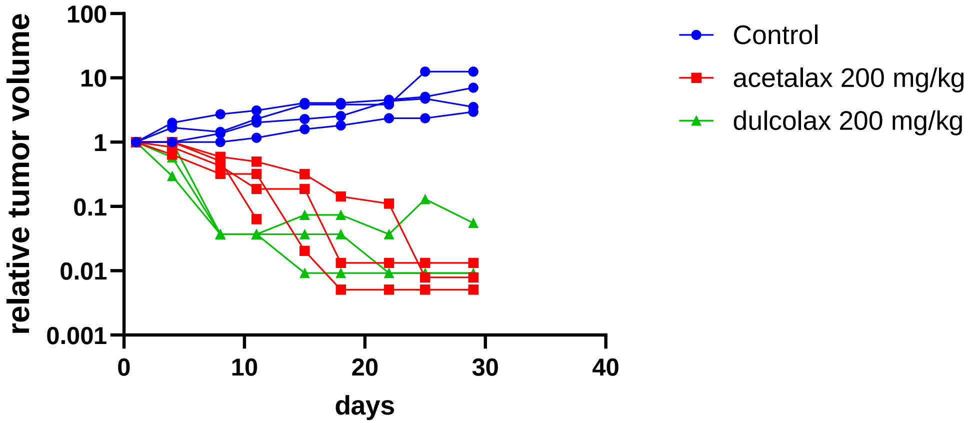

Dunn's multiple comparisons test	Mean rank diff.	Significant?	Summary	Adjusted P Value
Control vs. acetalax 200 mg/kg	12.22	Yes	**	0.0022
Control vs. dulcolax 200 mg/kg	14.44	Yes	***	0.0002

HBCx-12A (individual xenografts curves)

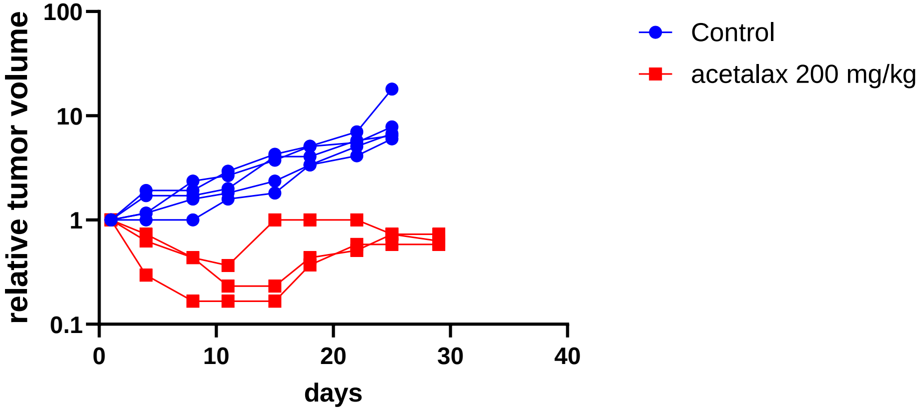

Column B	acetalax 200 mg/kg
vs.	vs.
Column A	Control
Mann Whitney test	
P value	0.0001
Exact or approximate P value?	Exact
P value summary	***
Significantly different (P < 0.05)?	Yes

HBCx-209 (individual xenografts curves)

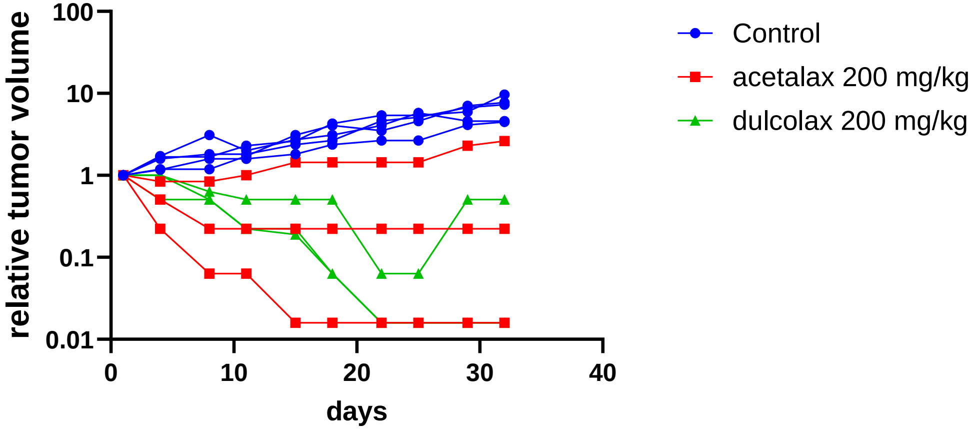

Dunn's multiple comparisons test	Mean rank diff.	Significant?	Summary	Adjusted P Value
Control vs. acetalax 200 mg/kg	11.80	Yes	**	0.0054
Control vs. dulcolax 200 mg/kg	17.90	Yes	****	<0.0001

Legend: *In vivo* response to Acetalax and/or bisacodyl in patient derived xenograph (PDX) models of TNBC. Acetalax or bisacodyl administered at 200 mg/kg in the HBCx-204 PDX model. Acetalax or bisacodyl administered at 200 mg/kg in the HBCx-209 PDX model. Acetalax administered at 200 mg/kg in the HBCx-12A PDX model. The y-axis units are log10.
