# Supplementary material for: Acetalax and Bisacodyl for the Treatment of Triple-Negative Breast Cancer: A Combined Molecular and Preclinical Study
Source: Cancer Res Commun. 2025 Feb 28;5(2):375–88. doi: 10.1158/2767-9764.CRC-24-0435 (PMC11869203; doi:10.1158/2767-9764.CRC-24-0435)
Supplement: Supplementary Figure 4 — Scatter plots of gene transcript levels versus acetalax activity for genes from the Figure 6 multivariate analysis. [file crc-24-0435_supplementary_figure_4_suppsf4.pdf]

Supplemental Figure 4

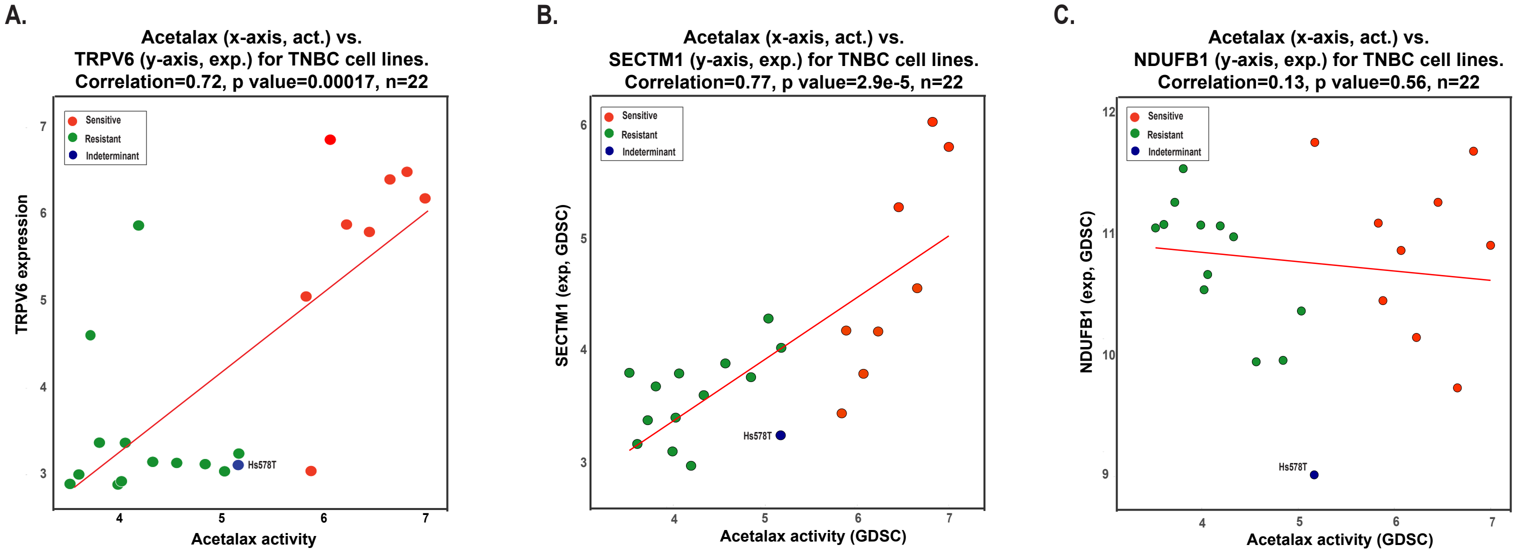

Legend: Scatter plots of gene transcript levels versus acetalax activity for genes from the Figure 6 multivariate analysis.
A. Acetalax activity (x-axis) versus TRPV6 transcript level (y-axis). **B.** Acetalax activity (x-axis) versus SECTM1 transcript level (y-axis). **C.** Acetalax activity (x-axis) versus NDUFB1 (y-axis). The transcript levels were measured by GDSC microarray. The circles are cell lines. The red lines are regression lines. "vs" is versus, "exp." is expression, and TNBC is triple negative breast cancer.
