# Supplementary material for: Acetalax and Bisacodyl for the Treatment of Triple-Negative Breast Cancer: A Combined Molecular and Preclinical Study
Source: Cancer Res Commun. 2025 Feb 28;5(2):375–88. doi: 10.1158/2767-9764.CRC-24-0435 (PMC11869203; doi:10.1158/2767-9764.CRC-24-0435)
Supplement: Supplementary Figure 5 — Tumor mass response as compared to untreated transcript expression. [file crc-24-0435_supplementary_figure_5_suppsf5.pdf]

Supplemental Figure 5: Biomarker analysis, PDXs

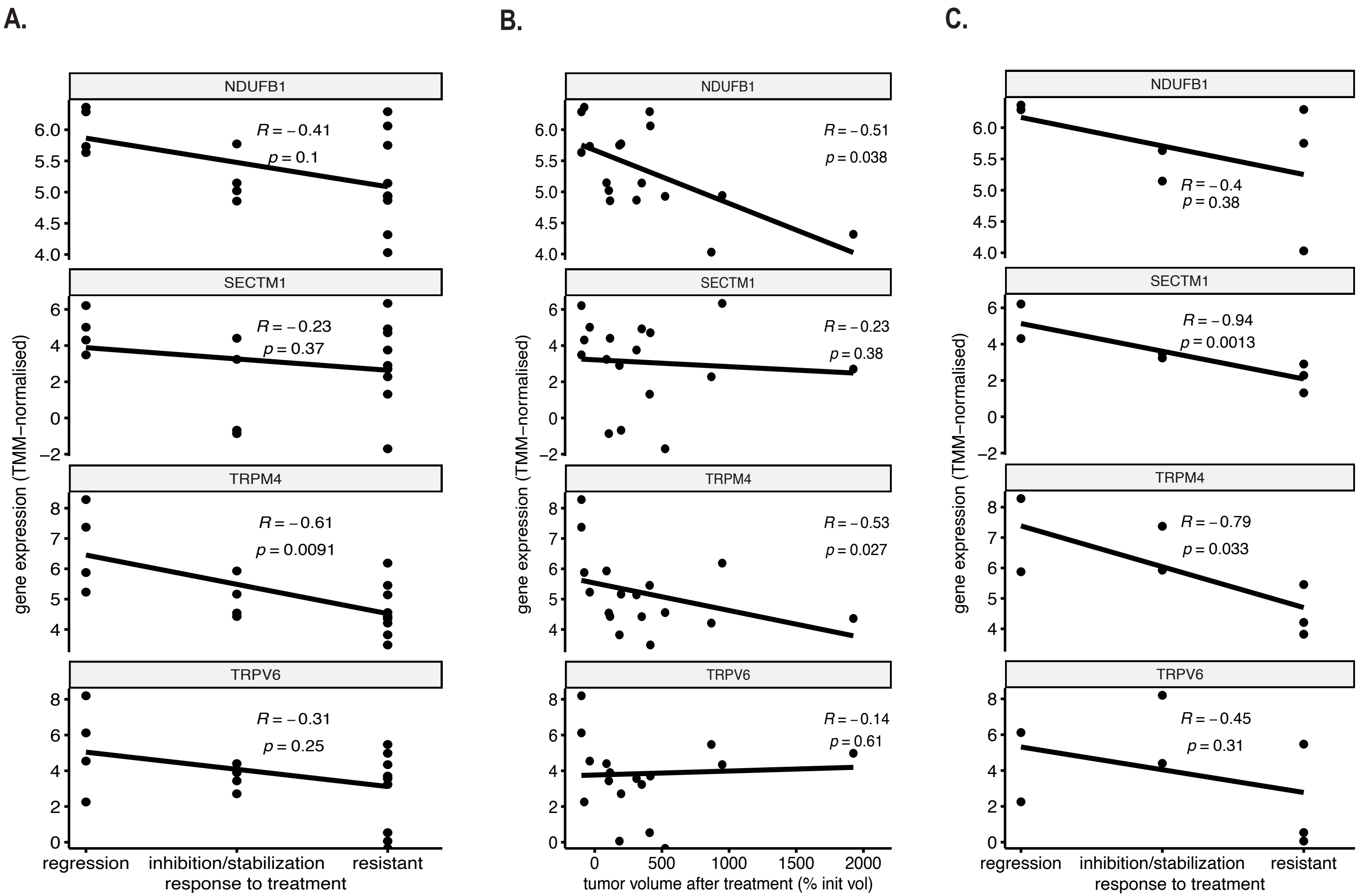

Legend. Tumor mass response as compared to untreated transcript expression.

A. Tumor mass following acetalax treatment as broken into three discreet categories (resistant, inhibition or stabilization and regression) as defined in Figure 3B compared to transcript levels of the four genes as measured by RNAseq. The x-axis is the three discreet categories, resistant, inhibition or stabilization and regression. The y-axis is the log2 transcript level as measured by RNAseq. **B.** Tumor mass following acetalax treatment as defined using the continuous values in Figure 3B compared to transcript levels of the four genes as measured by RNAseq. The x-axis is the continuous tumor volume (as a percentage of tumor volume upon treatment initiation). The y-axis is the log2 transcript level as measured by RNAseq. **C.** Tumor mass following bisacodyl treatment as broken into three discreet categories (resistant, inhibition or stabilization and regression) as defined in Figure 3E compared to transcript levels of the four genes as measured by RNAseq. The x-axis is the three discreet categories, resistant, inhibition or stabilization and regression. The y-axis is the log2 transcript level as measured by RNAseq.

Supplemental Figure 5: Biomarker analysis, PDXs
