# Supplementary material for: Acetalax and Bisacodyl for the Treatment of Triple-Negative Breast Cancer: A Combined Molecular and Preclinical Study
Source: Cancer Res Commun. 2025 Feb 28;5(2):375–88. doi: 10.1158/2767-9764.CRC-24-0435 (PMC11869203; doi:10.1158/2767-9764.CRC-24-0435)
Supplement: Supplementary Figure 6 — Scatter plots of ErSO drug activity versus acetalax activity and TRPM4 gene transcript levels. [file crc-24-0435_supplementary_figure_6_suppsf6.pdf]

Supplemental Figure 6

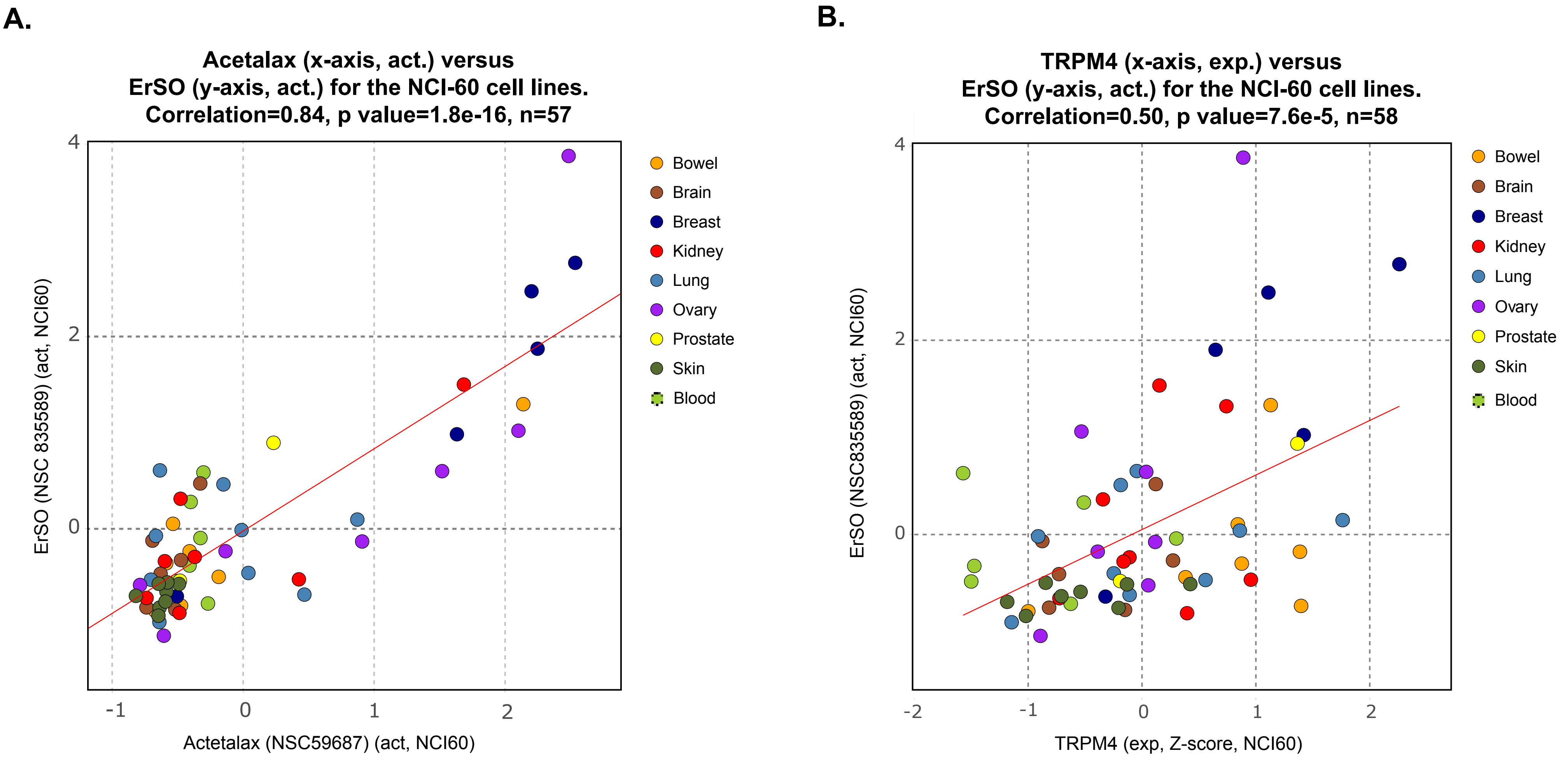

Legend. Scatter plots of ErSO drug activity versus acetalax activity and TRPM4 gene transcript levels.
A. Acetalax (x-axis) versus ErSO (y-axis) activity levels. **B.** TRPM4 transcript (x-axis) versus Acetalax activity levels (y-axis). The transcript levels were measured by NCI-60 microarray. The circles are cell lines. The red lines are regression lines. “exp.” is expression and “n” is the number of cells.
